# Current Updates on Cancer-Causing Types of Human Papillomaviruses (HPVs) in East, Southeast, and South Asia

**DOI:** 10.3390/cancers13112691

**Published:** 2021-05-30

**Authors:** Chichao Xia, Sile Li, Teng Long, Zigui Chen, Paul K. S. Chan, Siaw Shi Boon

**Affiliations:** Department of Microbiology, The Chinese University of Hong Kong, Hong Kong SAR 999077, China; cc_choice@link.cuhk.edu.hk (C.X.); lisile@link.cuhk.edu.hk (S.L.); jerrylongt@link.cuhk.edu.hk (T.L.); zigui.chen@cuhk.edu.hk (Z.C.); paulkschan@cuhk.edu.hk (P.K.S.C.)

**Keywords:** HPV, cervical cancer, head and neck cancer, E6, E7, diagnosis, HPV vaccine, pap smear, p16, p53

## Abstract

**Simple Summary:**

Among the over 200 human papillomavirus (HPV) genotypes identified, approximately 15 of them can cause human cancers. In this review, we provided an updated overview of the distribution of cancer-causing HPV genotypes by countries in East, Southeast and South Asia. Besides the standard screening and treatment methods employed in these regions, we unravel HPV detection methods and therapeutics utilised in certain countries that differ from other part of the world. The discrepancies may be partly due to health infrastructure, socio-economy and cultural diversities. Additionally, we highlighted the area lack of study, particularly on the oncogenicity of HPV genotype variants of high prevalence in these regions.

**Abstract:**

Human papillomavirus (HPV) infection remains one of the most prominent cancer-causing DNA viruses, contributing to approximately 5% of human cancers. While association between HPV and cervical cancers has been well-established, evidence on the attribution of head and neck cancers (HNC) to HPV have been increasing in recent years. Among the cancer-causing HPV genotypes, HPV16 and 18 remain the major contributors to cancers across the globe. Nonetheless, the distribution of HPV genotypes in ethnically, geographically, and socio-economically diverse East, Southeast, and South Asia may differ from other parts of the world. In this review, we garner and provide updated insight into various aspects of HPV reported in recent years (2015–2021) in these regions. We included: (i) the HPV genotypes detected in normal cancers of the uterine cervix and head and neck, as well as the distribution of the HPV genotypes by geography and age groups; (ii) the laboratory diagnostic methods and treatment regimens used within these regions; and (iii) the oncogenic properties of HPV prototypes and their variants contributing to carcinogenesis. More importantly, we also unveil the similarities and discrepancies between these aspects, the areas lacking study, and the challenges faced in HPV studies.

## 1. Introduction

Papillomaviruses, belong to the family of Papillomaviridae, are small and non-enveloped viruses of 52–55 nm in diameter. The virion contains a double stranded circular DNA genome that generally encodes six “Early” (E) genes, two “Late” (L) genes, and a long control region (LCR) or upper regulatory region (URR).

The human papillomavirus (HPV) “Early” open reading frames (ORFs) contain E1, E2, E4, E5, E6, and E7, all of which play pivotal roles during viral replication and tumourigenesis. E1 functions as an ATP-dependent viral DNA helicase [1], whilst E2 regulates transcription, initiates DNA replication, and partitions the viral genome [2]. E4, which is primarily expressed as an E1^E4 fusion protein, is involved in genome amplification, virus synthesis, release, and transmission [3,4]. HPV encodes for two major (E6 and E7) and one minor (E5) oncoproteins. E6 and E7 proteins degrade tumour suppressors p53 [5,6] and retinoblastoma pRB [7], respectively, in a proteosome-dependent manner. The E5 protein is a small transmembrane protein that can activate the epidermal growth factor (EGF) and platelet-derived growth factor (PDGF) receptors, as well as facilitate immune evasion [8]. The combinatorial action of HPV oncoproteins leads to elevated cell survival signalling, dysregulated cell cycles, and apoptotic checkpoints, hence allowing HPV to deploy host cell machineries to maintain viral replication and promote cancer progression.

The HPV “Late” (L) ORFs encode for the major (L1) and minor (L2) capsid proteins. Since L1 ORF is the most conserved PV gene, it is used for phylogenetic classification and for HPV vaccine production [9]. L2 plays a role in viral assembly and trafficking. Though the L2 protein lacks the capacity to spontaneously assemble as VLPs, it co-forms with L1 to enhance VLP assembly [10]. To date, there have been over 200 HPV genotypes identified that mainly infect the cutaneous or mucosal epithelia surface of skins, oral, or genital sites [11,12]. The HPVs have been categorized into five highly divergent genera—*Alpha, Beta*, *Gamma*, *Mu*, and *Nu* [12,13]. The oncogenic mucosal or so-called high-risk (hr) HPVs are in the *Alpha*-PV group. The high-risk HPVs (hrHPVs) contribute to cancers of the uterine cervix, head and neck, vulva, anus, and penis. Meanwhile, skin and oral cavity swabs harbour abundant *Beta-* and *Gamma*-PVs, which suggests that the divergence of HPVs followed prior adaptation in specific niches [14,15,16].

## 2. HPV Genotype Distribution in East, Southeast, and South Asia

East, Southeast, and South Asia encompass individuals of different ethnicities and genetic backgrounds who live in environmentally and socio-economically diverse countries. Due to these diversities and the discrepancies in study sample size and detection methods used in different studies, the reported range of HPV-positive rates remains large. The overall HPV-positive detection rate in normal women ranges from 7.2% (Malaysia) to 41.7% (Hong Kong). The HPV genotypes commonly detected in these regions are HPV16, 18, 52, 58, 31, 33, 35, 39, 45, 51, 56, 59, 68, 6, 11, 42, 43, and 81 [17,18,19,20,21,22,23,24,25,26,27,28,29,30,31,32,33,34,35,36,37,38,39,40,41,42,43,44,45,46,47,48,49,50,51,52,53,54,55,56,57,58,59,60].

Globally, the most prevalent hrHPV genotypes detected in human cancers are HPV16 (15.56–83.78%) and 18 (3.4–41.1%). The other most prevalent hrHPV genotypes include HPV31 (1.37–8.89%), HPV33 (0.74–9.1%), HPV35 (0.5–3.2%), HPV39 (0.7–13.33%), HPV45 (0.8–9.1%), HPV51 (0.3–18.8%), HPV52 (1.08–40.74%), HPV56 (0.2–9%), HPV58 (1.9–15.6%), HPV59 (0.6–4.4%), and HPV68 (0.4–11.11%) [56,61,62,63,64,65,66,67,68,69,70,71,72,73,74,75,76,77,78,79,80]. The HPV detection rate was also found to increase as lesions progress from precancerous (low-grade squamous intraepithelial (LSIL) at 48.12–91.5% and high-grade squamous intraepithelial lesion (HSIL) at 75.6–99.5%) to cancerous (invasive cervical cancer (ICC) at 64.3–100%) [56,61,62,63,64,65,66,67,68,69,70,71,72,73,74,75,76,77,78,79,80]. The detailed prevalence of hrHPV genotypes detected in different stages of cervical lesions by regions and countries is summarized in Table 1.

The HPV genotypes detected in head and neck squamous cell carcinoma (HNSCC) might differ from those in cervical cancers. HPV16 remains the most prevalent detected type (1.6–60.7%), followed by HPV31 (1.6–79.2%), HPV18 (0.7–15.1%), HPV56 (0.5–15.1%), HPV52 (2%), HPV33 (1.2%), and HPV35 (0.5%) [81,82,83,84,85,86,87,88,89,90,91,92,93,94,95,96,97,98,99,100,101,102]. Intriguingly, HPV31 (79.2%), HPV45 (87.4%), and HPV68 (49.1%) have been reported to have particularly high rates in Singapore. Thus far, there has been no report on the detection of HPV39, 51, 58, and 59 in HNSCC. Similar to cervical cancer, hrHPV positivity also increases as tumours progress from the low to high tumour, node, and metastasis (TNM) stages. This is summarized in Table 2. In the following section, we reveal the pattern of HPV genotype distribution reported in East, Southeast, and South Asia by countries, lesions, and age groups.

### 2.1. East Asia (China, Hong Kong SAR, Macao, Taiwan, South Korea, Mongolia, and Japan)

As China is a geographically wide country, we collated studies on the distribution of HPV genotypes according to traditional geographical regions: East, South, Southwest, Northwest, Central, North, and Northeast China. In general, the most prevalent hrHPV genotypes detected in normal uterine cervixes were found to be HPV16, 52, and 58, followed by HPV18, 31, 33, 35, 39, 45, 51, 56, 59, and 68 [17,18,19,20,21,22,23,24,25,26,27,28,29,30,31,32,33,34,35,36,38,39,40,41,42,43,44,45,63]. The most commonly detected low-risk Human Papillomavirus (lrHPV) types were found to be HPV6, 11, and 81. Based on age groups, HPV detection rate was shown to be the highest among women of age >55, followed by 30–55 years old, and <30 years old [17,18,19,20,21,22,23,24,25,26,27,28,29,30,31,32,33,34,35,36,38,39,40,41,42,43,44,45,63].

The hrHPV genotypes that are most frequently detected in cervical lesions are HPV16, 18, 31, 33, 35, 39, 45, 51, 52, 56, 58, 59, and 68 [61,62,63,64,65,66,67,68,69,70,71]. As previously mentioned, the HPV-positive rate has been found to increase as lesions progress from low grade (LSIL) (78.2–85.8%) and HSIL (75.6–100%)) to high grade (ICC (64.3–100%)) lesions. The most detected hrHPV genotypes in LSIL were found to be HPV58, 52, 51, 16, and 56, whereas the most detected hrHPV genotypes in HSIL were found to be HPV16, 58, 52, 51, and 33. The detection of HPV16, 18, 58, 52, and 51 as shown to be high in ICC. Meanwhile, in HNSCC, HPV16 was shown to be the most prevalent HPV genotypes detected, followed by HPV18 and HPV52 [26,83,86,104].

In Japan, South Korea, and Mongolia, the three most prevalent hrHPV types detected in normal cervical samples were shown to be HPV16, 18, and 31 [46,47,48,49,50,51]. Other commonly detected hrHPV types were HPV33, 35, 39, 45, 51, 52, 56, 58, 59, and 68. Meanwhile, HPV16, 52, 51, 18, and 58 were reported as the most prevalent types detected in cervical lesion samples [72,73,74]. Unfortunately, there are no data about HNSCC from these countries. The HPV genotypes detected in normal cervical samples and different age groups are summarized in Table 3.

### 2.2. Southeast Asia (Thailand, Vietnam, Malaysia, Singapore, Laos, the Philippines, and Indonesia)

In normal cervical samples, HPV16 and 18 are the most prevalent types detected, followed by HPV52 and 58 [52,53,54,55,56,57]. Other hrHPV and lrHPV (HPV31, 33, 35, 39, 45, 51, 56, 59, 68, 6, 11, 42, 43, and 81) types have also been detected, though at a relatively lower rate of less than 2%. Additionally, the HPV prevalence in women aged 30–55 years was found to be higher than that of women of <30 and >55 years old [52,53,54,55,56,57].

In cervical lesions, the detection of HPV16, 18, 31, 33, 35, 39, 45, 51, 52, 56, 58, 59, and 68 has been common [56,75,76]. Intriguingly, among the countries included in this review, Laos reported the lowest HPV-positive rate in cervical lesions [76]. In HNSCC, it is worth noting that HPV31, 45, and 68 were found to be of high prevalence in Singapore [85]. In TNM staging for HNSCC, the overall HPV positivity was reported to have increased from T1 to T4 (18.8–100%) and from N0 to N3 (11.5–60%). The distribution of HPV genotypes in different age groups in Southeast Asia is shown in Table 4.

### 2.3. South Asia (India, Bangladesh, Sri Lanka, Nepal, Bangladesh, and Bhutan)

The pattern of HPV genotypes detected in South Asia differs from East and Southeast Asia. The ranking of HPV genotypes detected in normal cervical samples was revealed to be HPV16, HPV39, 58, 33, and 18 [59,60,105]. Other HPVs were also detected, though at a lower rate of less than 2%. Among countries in the region, India reported the highest cases of ICC, followed by Bangladesh, Pakistan, and Nepal [106,107]. For the cases of single HPV infection in cervical cancer, HPV16 remains the most detected type, followed by HPV18 and 45 [108,109]. The positive HPV detection rate has been reported to be the highest among women aged >55 (12.9–30%), followed by women of 30–55 and <30 years old [59,60,105].

For HNSCC samples with a single HPV infection, most of the samples have been found to be positive for HPV16. Other detected hrHPV types include HPV31, 18, 56, 52, 33, and 35 [84,87,88,89,90,91,92,93,94,95,96,97,98,99,100,101,102]. Additionally, samples detected with HPV16/18 co-infection and multiple HPV genotypes were also reported [110]. When dissecting HPV positivity by TNM staging, hrHPV positivity was demonstrated to have increased from T1 to T4 (0–100%), as well as from N0 to N3 (0–60%). These data clearly showed the contribution of HPV to the malignant progression of HNSCC. Meanwhile, there have been no epidemiology data on HPV from Maldives in the last five years. HPV prevalence by genotypes and age groups in South Asia is summarized in Table 5.

## 3. HPV Screening

Screening is an essential way to detect and control HPV infection. To date, various guidelines on the screening of HPV have been published, including the World Health Organization (WHO) HPV Laboratory Manual [111]. However, the implementation of a uniform and standardized screening method within a country can be challenging. Diagnostic laboratories could just use a cytology test, like the Papanicolaou (Pap) test, or combine it with an HPV nucleic acid test as the primary screening method. A triage HPV test, which combines cytology, an HPV nucleic acid test, and the immunostaining of tissues with p16/Ki67 surrogate markers, has also been widely employed as a screening method. The latter method was outlined in the 2014 WHO (Fourth Edition) classification criteria [112,113]. However, in low- and middle-income countries, due to the lack of financial support and establishment for proper health infrastructure, the implementation of the triage HPV test can be burdensome. Instead, these countries may adopt visual inspection after the application of acetic acid (VIA) as the primary screening strategy [114]. Fortunately, developed countries like Japan, France, the Netherlands, and the WHO have collaborated and provided assistance to countries with limited resources for HPV epidemiological studies.

### 3.1. Sampling Methods

Sample collection methods are clearly described in the WHO HPV Laboratory Manual, and they have been widely adopted by many countries within the region. However, the sampling tools used by health practitioners may depend on anatomical sites, the availability of medical supplies, and resources. In general, exfoliated tissues from uterine cervix, skin, and other anogenital areas are collected using swabs/brushes made of nylon, polyester, or cotton. The samples are then stored in preservation transport media. Example of used swabs/brushes include iCleanhcy flocked swabs (Huachenyang Corporation, Shenzhen, China), FLOQSwab R100 and FLOQSwab U80 (Copan Diagnostics Inc., Murrieta, CA, USA) [115,116], and Dacron^®^ polyester swabs [117]. Samples can be preserved in transport media like phosphate-buffered saline (PBS) [118], PreservCyt solution (Hologic Corporation, Marlborough, MA, USA) [116,119], LiquiPrep preservation solution (LGM International Inc., Fort Lauderdale, FL, USA) [115], and Specimen Transport Medium (Qiagen, Hilden, Germany) [117]. For the detection of HPV DNA in the head and neck (HNC) region, oral swabs and rinse are commonly kept in saline [120].

In recent years, self-sampling has become popular, partly due to convenience and cultural conservation. Another benefit to self-collection is that the HPV-positive detection rate in tissue, ranging from dysplasia to high grade lesions, is comparable to that collected by medical professionals, as shown by studies conducted in Thailand, Hong Kong, and mainland China [121,122,123]. In addition to self-collected first-void urine [124,125,126], self-sampling kits for the Pap test and the detection of HPV DNA have also been made available for collection at designated health centres or posted via mail [123]. It would be worth implementing the collection of self-sampling kits in local pharmacies, online personal care stores, convenient, or departmental stores. Upon collection, one could post the sample to designated laboratories and track test result via a mobile application. In India, a research group adopted an interesting way to collect samples from the uterine cervix by collecting used menstrual pads (either home-made or commercial available) [127,128]. This method was deemed accurate and able to provide comparable results to samples collected by gynaecologists [125,128]. However, this may only hold true when one highly sensitive and specific test is used. In advanced conditions, one should refer to a gynaecologist for sample collection and subsequent clinical management.

### 3.2. Cytological and Histological Assessment

The detection of abnormal cervical tissues using the Pap test, a multichromatic cytological stain, has been widely adopted by diagnostic laboratories for decades. Nonetheless, the Pap test does not discern whether the changes in cell morphology and proliferation pattern are due to HPV infection. A cost-effective way to make this discernment is by performing the triage HPV test [129].

### 3.3. HPV Nucleic Acid Detection

HPV has adopted an intelligent way to replicate, along with host cell differentiation and maturation, without eliciting host cell lysis and robust immune response. In addition, the transient HPV infection makes HPV serological test inaccurate. Therefore, a sturdy way to detect HPV is by detecting viral nucleic acids, mainly through targeted polymerase chain reaction (PCR) using consensus or type specific primer pairs. Commonly used primer pairs include the standard PGMY09/11 or GP5+/GP6+ L1 consensus primers [127,128,130,131] and PCR primers targeting HPVE6/E7 [52,112,116,132,133,134] or E6*I [85], both of which are followed by HPV genotype discrimination using Sanger sequencing [55,101,135,136,137]. Many laboratories have also employed the PGMY-CHU assay [138,139] which is outlined in the WHO HPV Laboratory Manual [111], in which the HPVL1 that is amplified using PGMY09/11 primers is detected via reverse blotting hybridization. Additionally, HPV mRNA and signal amplification can be detected via liquid-phase or in situ hybridization methods, as applied in Hybrid Capture 2 (HC2) (Qiagen Gaithersburg, MD, USA) [85]. Other validated commercial HPV tests used across the region include the Cervista HPV HR Test (Hologic, Madison, WI, USA) [140], careHPV Test (Qiagen Gaithersburg, MD, USA) [140], Linear Array (Roche Molecular Systems Inc., Pleasanton, CA, USA) [49,141,142,143], Cobas 4800 HPV Test (Roche Molecular Systems Inc., CA, USA) [140,144], Xpert HPV (Cepheid, CA, USA) [145], INNO-LiPA HPV Genotyping (Fujirebio, Gent, Belgium) [146,147,148,149], and Luminex Genotyping GP HR (Diassay, Netherlands) [150]. Additionally, there are wide varieties of commercial HPV genotyping kits that can simultaneously detect more than 20 HPV genotypes. However, the vast majority of these kits are not clinically validated according to the Meijer guidelines [150]. These commercial detection systems are summarized in Table 6. In the following section, we reveal the HPV nucleic acid detection platforms and in-house methods used in East, Southeast, and South Asia.

#### 3.3.1. East Asia

China the country that produces most of the commercial HPV nucleic acid test kits in the world [150]. In general, the majority of the HPV kits cover the detection of the most prevalent HPV genotypes, including 13 hrHPV (HPV16, 18, 31, 33, 35, 39, 45, 51, 52, 56, 58, 59, and 68), two intermediate-risk subtypes (66 and 53), and six lrHPV (HPV6, 11, 42, 43, 44, and CP8304 or equivalent to HPV81). Most of the kits utilize the high-throughput gene chip-PCR-fluorescence technique, flow-through hybridization, PCR or real-time PCR, and reverse dot blot (RBD) technologies. Available commercial kits include the HPV GenoArray Diagnostic Kit (Hybribio, Chaozhou, China) [151,152,153,154,155], HPV Geno-Array test kit (Genetel Pharmaceuticals Co, Ltd., Shenzhen, China) [119,158] (US Patents 5,741,647 and 6,020,187), HPV Genotyping Panel Kit (Tellgen Life Science Co. Ltd., Shanghai, China) [159], HPV Genotyping Detection Kit (Crystal Core^®^, CapitalBio Corporation, Beijing, China) [59,160], HPV Nucleic Acid Amplification Typing Test Kit (Kaipu Biochemical Company, Guangdong, China) [121], HPV Genotyping Kit (bioPerfectus Technologies, Jiangsu, China) [148], PCR-RBD assay (Yaneng Bioscience, Shenzhen, China) [33,83,127,155,161,162], HPV Genotyping Real Time PCR kit (Zhejiang Bio-Tech Co. Ltd., Shanghai, China) [62,163,164], SNIPER^®^ HPV Genotyping Diagnosis Kit (Genetel, Shenzhen, China) [131], and GenoFlow Human Papillomavirus Array Test (DiagCor Bioscience Inc., Hong Kong SAR, China) [173]. In addition to these locally produced detection kits, SPF10 PCR-DEIA-LiPA25 (Labo Biomedical Products, Rijswijk, The Netherlands), an HPV detection platform that combines a DNA enzyme immunoassay (DEIA), reverse hybridization, and multiplex-type-specific PCR has also been used [165]. This last method is highly sensitive and specific despite its relatively lengthy and sophisticated procedures.

In Hong Kong, our team established a joint effort with a research team in Macao to study HPV prevalence. We utilised modified GP5+/6+ L1, designated as GP5+/GP6+_52HK primers, for the better detection of HPV52 variants of high prevalence in East Asia. We also performed HPV genotyping in oral rinse samples using the cutting-edge next generation sequencing (NGS) [166]. Other used diagnostic methods include tissue immunostaining with p16, a droplet digital PCR (ddPCR) Supermix containing E7 and L1 HPV genotype-specific probes [120]. In Taiwan, a locally developed EasyChip HPV genotyping array (King-Car Co. Ltd., Yilan, Taiwan) [167,168] is available for HPV genotyping using a PCR-based RDB platform.

In South Korea, high-throughput HPV genotyping using chip-based technology appears to be preferred in recent years. For instance, the MyHPV Chip Kit^®^ (BioMedLab, Seoul, Korea) [169], HPV Genotyping Chip^™^ Kit (AGBIO Diagnostics, Seoul, Korea) [148], and Cheil HPV DNA Chip Kit (Cheil General Hospital, Seoul, Korea) [72] were developed to detect 24, 32, and 36 clinically important HPV genotypes, respectively. Some studies used two different HPV detection platforms, e.g., an HPV Liquid Bead Microarray (Osang Healthcare, Anyang, China) and a qRT-PCR based Anyplex™ II HPV28 (Seegene, Seoul, Korea) in [47] and an HPV 9G DNA chip (Biometrix Technology Inc., Chuncheon, Korea) and GeneFinder HPV Liquid Bead MicroArray Genotype kit (GeneFinder; Infopia Inc., Anyang, China) in [170].

In Japan, Genosearch-31 (GS-31) (MBL, Nagoya, Japan), a high-resolution PCR-SSOP-Luminex-based method that combines PCR and a sequence-specific oligonucleotide probe (SSOP) with a Luminex xMAP system has been used to discriminate 31 HPV genotypes in various studies [51,171,172]. Japanese research teams have also collaborated with Mongolia, Cambodia, and Laos in HPV epidemiological studies. In these studies, the clinical samples collected by local gynaecologists were sent to laboratories in Japan for analyses using validated methods like the Linear Array HPV Genotyping Test (Roche Molecular Systems, Branchburg, NJ, USA) [49] and PGMY-CHU assay [139], as well as an in-house designed methods to identify fragments of restriction-enzyme-digested, HPV genotype-targeted PCR amplicons [76].

#### 3.3.2. Southeast Asia

Commercial kits run on different detection platforms, including the real-time PCR (AmoyDx^®^ Human Papillomavirus Genotyping Detection Kit from Amoy Diagnostics, Xiamen, China) [174], gene chip systems (HPV GenoArray Diagnostic Kit from Hybribrio, Chauzhou, China) [115,156], flow cytometric assays (HPV OncoTect^®^ E6, E7 mRNA Kit, incellDx, CA, USA) [176], PCR-Restriction Fragment Length Polymorphism (PCR-RFLP) assay [53], microplate colorimetric hybridization assay (MCHA) [115,179], GenoFlow HPV Array Test Kit (DiagCor Bioscience, Hong Kong) [54], and Anyplex II HPV28 Detection Kit (Seegene, Seoul, Korea) have been used [157].

In Thailand, prior to using commercial kits for HPV genotyping, Nopmaneepaisarn and colleagues used a DNA ISH HPV III Family Probe (Ventana Medical Systems, Tucson, AZ, USA) to identify hrHPV. This method can detect episomal and integrated viral genome expression [174]. In-house HPV detection methods [153,175,177], such as those described by van de Brule et al. [180] and Waterboer et al. [181], have also been employed. These studies, respectively, utilised high-throughput RBD and a multiplex serology assay for HPV genotype discrimination. On the other hand, Sanger sequencing [115], in-house nested-PCR [52], and fluorescence-based PCR-hybridization array [103] have also been used. A recent study suggested the feasibility of using nanotechnology to detect HPV DNA. This could be an experimental diagnostic tool in the future [182,183].

#### 3.3.3. South Asia

In addition to following the standard and validated assays for HPV screening, various laboratories also use in-house PCR-based [184,185] and commercial kits for HPV screening. An Indian team adopted a fluorescence-based PCR assay developed by Schmitt and colleagues in IACR (Lyon, France). They used a type-specific E7 PCR bead-based multiplex assay to identify 22 HPV genotypes [178,186]. Commercial kits produced abroad have also been used, including the PapType hrHPV detection and genotyping kit (Genera Biosystems Limited, Australia) [60], HPV GenoArray kit (Hybribrio, Chauzhou, China) [59], and Anyplex™II HPV28 Detection kit (Seegene Inc., Seoul, Korea) [105]. Alternatively, clinical samples can be sent to a diagnostic service laboratory abroad or to collaborators for HPV screening [80,187]. For instance, clinical samples collected b ay local team in Bhutan were analysed in the Infections Section Laboratory at IARC, Lyon [126,188,189].

Some of the non-validated commercial HPV detection kits assess the sensitivity and specificity of their products via comparisons to validated assays. For instance, when comparing PapType HPV assay to Hybrid Capture 2 (HC2) and Linear Array (LA) HPV tests, LA (91.6%) was found to be more sensitive than PapType (90.3%) and HC2 (79.8%), while the specificity of HC2 (55.3%) was found to be higher than PapType (52.5%) and LA (51.7%) [190]. The HPV GenoArray Test Kit (Hybribio, Chauzhou) has been claimed to perform better in detecting HPV52 than LA [158], and the GeneFlow HPV array test (Diagcor Bioscience, Hong Kong) is comparable to LA [173]. Despite the ability to detect more HPV genotypes offered by the aforementioned, non-validated HPV test kits, their sensitivity and accuracy might be compromised when compared to standard assays [191] and should be further validated using more clinical samples.

## 4. Treatment Regimens

In the advanced medical world, various anti-cancer drugs are available to treat cancers, including cancers caused by HPV. Unfortunately, none of these anti-cancer drugs are tailored for HPV-associated cancers. In this part, we describe the treatment regimens for HPV-associated cancers employed within South, East, and Southeast Asia, most of which are standardized and well-established treatment options, including HPV vaccines, surgical ablation, photodynamic therapy, radiotherapy, chemotherapy, immunotherapy, and anti-viral agents. However, certain countries may use locally produced drugs to tackle the disease.

### 4.1. HPV Vaccines

The implementation of HPV vaccination programs has curtailed the incidence of HPV-associated cancers successfully, particularly in HPV-naïve individuals. The US Food and Drug Administration (FDA) has approved HPV prophylactic vaccines, including Gardasil, Cervarix, and Gardasil^®^9, that been shown to be plausibly effective in lowering the risk of HPV infection and providing immune protection in both women and men [192]. Results from the VIVIANTES study, a randomized controlled Phase III clinical trial, revealed the 90% efficacy of an HPV16/18 ASO_4_-adjuvant vaccine (Cervarix, GSK, Belgium) in providing immune protection for HPV16/18, as well as at least 65% cross-protection for HPV31 and 45 [193]. In China, Phase II and III clinical trials revealed that an HPV16/18 recombinant bivalent vaccine (Xiamen Innovax Biotech, Xiamen, China) produced in *Escherichia coli* provided economical and safe alternative immune-protection against HPV infection [194,195]. A South Korean team showed that an AcHERV-HPV L1 vaccine provided immune-protection for HPV16, 18, and 58. The vaccine also possessed anti-tumour properties to a better extent than that of Cervarix [196].

The exploration of HPVE6 and/or E7 DNA therapeutic vaccines has become increasingly popular. These vaccines possess several advantages over prophylactic vaccines. Phase I and II clinical trials have shown that they are able to stimulate a broad range of immune response in immunocompetent hosts, as well have possessing simplicity and low-cost production [197]. In an in vivo preclinical trial, the intravaginal injection and electroporation of an HPV E7–calreticulin chimera DNA vaccine alone or co-administered with interleukin-2 (IL-2) effectively elicited E7-specific cytotoxic T lymphocyte (CTL) response [198]. Even though this mode of administration appears to be less invasive than surgery and applicable in clinics [199], its wide implementation can be technically challenging. In contrast to the efficacy of HPV vaccines, controversies as to whether these vaccines can evoke sufficient host immune response to clear HPV infection, suppress viral persistence, and prevent HPV recurrence remain [200].

### 4.2. Surgical Removal

For precancerous cervical lesions, as recommended in WHO guidelines [201], many countries employ a “screen-and-treat” strategy. Following recommendations from specialists, the surgical removal of ICC can be performed. This includes the ablative surgery of solid and non-metastatic cancers executed via cryotherapy, laser ablation, electrofulguration, and cold coagulation, which can be achieved through cold knife conization (CKC), loop electrosurgical excisional procedure (LEEP), and/or hysterectomy [202]. Tumour ablation via cryotherapy offers a more-than 80% success rate [203]. Even though mere surgery may improve patient survival rate, a combination with interferon treatment would give a more promising outcome [114]. Nonetheless, the removal of a tumour does not promise the complete eradication of HPV and may result in recurrence [204,205,206]. This is largely due to viral persistence, especially that of HPV16 and 18 [206]. Several studies found that patients who take HPV prophylactic vaccine post-surgery had a reduced risk of recurrence [207,208]. However, HPV vaccination may not pose obvious effects on viral persistency [209]. The necessity to get an HPV vaccine post-surgery depends on whether the patient can afford the cost of vaccination, the health condition post-surgery, and the accessibility and availability of the vaccine. As outlined in the WHO guidelines, an HPV test should follow surgery, but this sequence can be reverted in low-income countries [201].

### 4.3. Photodynamic

Photodynamic therapy (PDT) serves as a treatment option when surgery is not recommended. PDT is an emerging non-invasive curative measure for HPV-associated cancers. Prior to PDT, the treatment of cervical warts or high-grade lesions with 5-aminolevulinic acid (ALA) [210] or polyhematoporphyrin ester/ester (PHE) [211] greatly reduces HPV viral load. Mechanistically, ALA-PDT works by inducing autophagy, thus reducing cell viability and proliferation [210]. Unfortunately, ALA-PDT can lead to prolonged erythema, pain, and *Staphylococcus sp.* infection. Remedies to this are treatment with topical fusidic acid or mupirocin [212].

### 4.4. Radiotherapy and Chemotherapy

HPV-bearing cancer cells are sensitive to radiation and prone to cell death stimuli. These issues make radiotherapy and chemotherapy effective in both HPV-associated cervical cancers and HNSCC. Generally, these treatment modalities have better impacts on the early stages of ICC. The common chemotherapeutics for both localized cervical cancer and HNSCC include docetaxel, cisplatin, and 5-fluorouracil [213,214].

Patients with locally advanced cervical cancer treated with an extended field of external-beam radiotherapy covering whole pelvis and extending to para-aortic lymph nodes may have an added advantage. This approach was found to better reduce mortality and para-aortic lymph node recurrence than pelvic radiotherapy/chemotherapy [215]. Meanwhile, for patients with recurrent cervical cancer within pelvic cavity post-hysterectomy, the majority of patients responded positively to salvage radiotherapy with or without concurrent chemotherapy. The regimen was tolerable and offered >60% five-year overall survival rate [216]. Patients with relapsed and advanced cervical cancer involving lymphatic metastasis were treated with radical surgery—salvage radiotherapy with concurrent chemotherapy (combination of docetaxel and cisplatin/cyclophosphamide, cisplatin and 5-fluorouracil, and paclitaxel and carboplatin) [213,217].

For HPV-related HNSCC, the prognosis and survival of patients at early stages are generally good, so chemotherapy may be not be required. Rather, transoral surgery or radiotherapy could be sufficient to curb disease progression and offer an elevated quality of life. In advanced stages of HPV-associated oropharyngeal squamous cell carcinoma (OPSCC), induction chemotherapy and chemoradiotherapy with cisplatin tends to be the choice of treatment [218]. A Japanese team showed that the HPV-associated OPSCC of TNM stage 1/2, radiotherapy alone, or concurrent chemotherapy resulted in 69.5% five-year recurrence-free survival and overall survival rates [218,219].

### 4.5. Immunotherapy

Immunotherapy has emerged as a pivotal treatment strategy for cancers, including HPV-associated cancers. The prime choices include immune checkpoint inhibitors targeting programmed cell death 1 (PD1)/ligand 1 (PDL1), like pembrolizumab and nivolumab. While HPV infection alone also promotes the infiltration of T cells into tumour sites [220], immunotherapy enhances the efficacy in eliminating cancer cells via a similar strategy. Clinical trials conducted in South Korea and China clearly unleashed a lower toxicity of pembrolizumab than platinum-based chemotherapeutics and cetuximab, an epidermal growth factor receptor inhibitor, and a higher efficacy in treating HPV-positive patients than -negative HNSCC patients [220,221,222]. Like pembrolizumab, a Phase II clinical trial in Japan showed that nivolumab was safe and able to reduce tumour size of cervical cancer [223]. Additionally, in CheckMate 141, a Phase III clinical trial conducted in 64 locations around the world including Hong Kong, Japan, South Korea, and Taiwan, showed that nivolumab significantly increased the two-year overall survival of patients with recurrent and metastatic HNSCC regardless of HPV status and PD-1 expression [224]. The clinical efficacy of ipilimumab, a monoclonal cytotoxic T-lymphocyte-associated protein 4 antibody, for HPV-associated cancer was tested in Canada and the US [225], but its clinical efficacy in East, Southeast, and South Asia has not been evaluated in recent years.

The efficacy of other immune modulators has also been explored. In South Korea, women were recruited to study the safety and efficacy of poly-gamma glutamic acid (γ-PGA) in treating CIN I cervical cancer and clearing HPV infection. This Phase IIb clinical trial revealed that the treatment enhanced the clearance of HPV [226]. In another Phase I/IIa clinical trial, Park and colleagues studied BLS-M07 in patients with CIN 3 cervical cancer. Basically, the treatment involved the oral administration of genetically modified *Lactobacillus casei*, a probiotic, with HPV16E7 expressed on its surface. Intriguingly, the treatment appeared to be safe and effective in 75% of patients, and it was able to provoke HPVE7-specific immune IgG production [227].

### 4.6. Antiviral Therapy

To date, the choice of HPV-specific antiviral agents is limited. Several studies have shown the clinical efficacy of antimicrobial agents in dampening the HPV viral life cycle and stimulating the host HPV-specific immune response. In a Phase I/IIa clinical trial, Jiang and colleagues showed that 3-hydroxyphthalic anhydride-modified bovine beta-lactoglobulin (JB01) topical treatment resulted in a marked reduction of HPV viral load in women with hrHPV infection [228]. Meanwhile, combination antiretroviral therapy (cART), which comprised five nucleoside analogue reverse transcriptase inhibitors (zidovudine, didanosine, zalcitabine, stavudine, and lamivudine), two non-nucleoside reverse transcriptase inhibitors (delavirdine and nevirapine), and four protease inhibitors (saquinavir, ritonavir, indinavir, and nelfinavir), was able to elicit HPVE6-specific immunological response with enhanced CD4 T cell count in HIV patients [229,230]. A Phase II clinical trial showed that the application of a proprietary antiviral agent, REBACIN^®^ cream (REBACIN Vaginal Gel; Hainan SR-Bio Pharma Co., Ltd., Hainan, China) onto intravaginal lesions could effectively suppress HPV oncogenes expression [231].

For treating an HPV-attributable benign condition, like recalcitrant warts and laryngeal papillomatosis, FIT039 (a dual cyclin-dependent kinase 9 (CDK9) inhibitor and an antiviral agent [232]) together with isotretinoin as an adjuvant therapeutic were promising in immunocompromised patients [233]. Additionally, the intralesional treatment of laryngeal papillomatosis with antiviral cidofovir was sufficient to improve disease severity [234]. Other potentially antiviral agents that showed efficacy in inhibiting the migration and tumour formation of HPV-positive cervical cancers in a preclinical study included ribavirin and indinavir [235]. However, further clinical validation should be done to confirm their anti-HPV specificity in clinics.

### 4.7. Other Potential Options: Non-HPV-Targeted Therapeutics, Natural Compounds, and Gene Silencing/Editing

Repurposing anti-cancer drugs can fast-tract the use of these drugs to treat HPV-associated cancers in clinics, e.g., ormeloxifene [236] and gefitinib [237]. Ormeloxifene is a non-hormonal and non-steroidal anti-oestrogen drug used to treat advanced stages of breast cancer, whereas gefitinib is a tyrosine kinase inhibitor. These drugs showed great efficacy in reverting cancer phenotypes and halting malignant progression induced by HPV.

In China, traditional medicines have been used to treat benign hyperplasia caused by HPV. For instance, a four-month treatment with a topical cream composed of paiteling (Beijing Paite Biotechnology Limited Company, Beijing, China) showed a 92% efficacy in reducing the abundancy of HPV40, 35, and 25 in condyloma acuminatum without recurrence [238]. The Baofukang suppository and Er Miao decoction also shown efficacy in decreasing the HPV viral load in cervical samples [239].

Some preclinical studies have unravelled the anti-tumour properties of natural compounds and traditional medicines using cell-based and animal models. Extracts from *Juglans mandshurica* (Manchuria walnut), juglone [220], and *Cudrania tricuspidata* stem [240] (the last of which is a traditional medicine commonly used in Korea), can inhibit the growth of HPV-positive cells, while *Pleurotus ferulae* polysaccharides (PFPS), a traditional Chinese medicine, works as an adjuvant in HPV dendritic cell-based vaccines and showed a great efficiency in reducing tumour volume in an in vivo model [241]. Intriguingly, numerous studies have also shown that essential ingredients in Asian cuisines possess anti-HPV properties. For instance, pure extracts from turmeric (*Curcuma longa*), neem (*Azadirachta indica*), tulasi or holy basil (*Occumum sanctum*), and ginger (*Zingeber officinale*) can instigate the activation of the apoptotic pathway in an in vitro system [242,243]. Additionally, flaxseed oil was able to downregulate HPV oncoprotein expression, thus restoring tumour suppressor expression and reducing tumour burden, in a mouse model [244]. Despite demonstrating great efficacy when using in vitro and non-human models, biological relevant preclinical and clinical models should be used to recapitulate their genuine efficacy as choices of treatment for HPV-associated cancers.

A short inhibitory RNA sequence that complementarily binds to the mRNA of HPV onco- and transcription activator-like effector nucleases (TALENs) [245], micro-RNAs like miR-214 [246], and iron-chelating drugs [247] showed a high efficiency in depleting the expression of HPV oncoproteins, thus reverting tumour phenotypes. These molecules require a coupling and delivery system into host cells in order to exert their functions. One of them used nanoparticles based on poly-β-amino ester (PBAE) to deliver HPV16E7-targeting CRISPR/short hairpin RNA (shRNA) in an in vivo model [248]. Though silencing or editing the expression of HPV oncogenes appears to be promising in in vitro and in vivo models, its journey in clinical trials might be bumpy.

## 5. Genetic Variations within E6 and E7 Oncoproteins Contribute to Their Differential Carcinogenicity

The vast majority of studies have focused on the oncogenic properties of HPV16 and 18, as well as 31 E6 and E7 prototypes. Various studies have also focused on identifying commonly circulating HPV variants in their locality. Through our search, laboratory evidence connecting the link between natural occurring amino acid changes of HPV oncoproteins to their mechanistic roles in promoting cancer progression is lacking. In East Asia, our team identified several HPV52 and 58 variants. Strikingly, we figured out that these variants carry amino acid mutations within E6 for HPV52 and within E7 in HPV58. In the following sections, we describe the impact of the amino acid mutations of HPV52 E6 and HPV58 E7 variants on their oncogenic properties.

### 5.1. HPV52 E6 Variants

Though HPV52 is the seventh most commonly detected HPV type worldwide [249,250], its ranking comes after HPV16 and 18 in East and Southeast Asia [251,252]. Based on our previous epidemiological findings, we discovered several genetic variations within E6, and these E6 variants were found to be highly associated with cervical cancer risk [253,254]. The three most commonly circulating HPV52 E6 natural variants are designated as V1 (K93R), V2 (E14D/V92L), and V3 (K93R/N122K) [255]. The V1 variant was indicated to be related to 98% of HPV52-positive cases in Japan [256]. Even though the V1 variant exhibited stronger colony formation and cell migration abilities than its prototype and other variants [257], these three variants degrade the p53 and PDZ proteins at similar levels [257]. Perhaps these variants target a subset of not-yet identified proteins that can induce cell growth in an anchorage-independent manner and migrate. Further proteomic and functional studies should be conducted to understand the molecular mechanism of how these HPV52 E6 variants contribute to enhanced carcinogenicity.

### 5.2. HPV58 E7 Variants

HPV58 is the second or third most prevalent HPV genotype detected in cervical cancer in East Asia [249,250]. Strikingly, our previous meta-analysis showed a higher attribution of HPV58 to cervical cancer, at an extent that was 3.7-fold higher than other part of the world [251]. We also discovered three common circulating natural variants of HPV58 E7: V1 (T20I/G63S), V2 (G41R/G63D) and V3 (T74A/D76E) [251]. Among these variants, V1 was found to possess a stronger epidemiological relation to cervical cancer risk [258]. We then decided to provide further experimental evidence to explain their relative contribution to cancer risk. Indeed, when comparing these HPV58 E7 variants, V1 showed a greater ability to induce the immortalization and transformation of primary cells [259], promoting cell proliferation, migration, invasion, and increased tumour burden in athymic nude mice [260]. We also provided a molecular explanation for this. V1 can degrade pRb, as well as activate AKT and K-Ras/extracellular signal-regulated kinase (ERK) signalling pathways, more effectively than its prototype and other variants [259,260]. This can potentially explain the high prevalence of HPV58 in cervical lesions and its increased association with cervical cancer risks.

## 6. Concluding Remarks

In low- and middle-income countries, the general population may have some grasp on cervical cancer. Their understanding about proper hygiene, the causal link between HPV and cancer, remains poor, and their knowledge about the HPV vaccine as a preventive measure is lacking [261,262]. In these regions, the implementation of a general healthcare system is challenging, and access to an HPV vaccine may appear to be a luxury. With the assistance provided by established laboratories in developed countries and the WHO, HPV screening programmes have been implemented. The number of subjects recruited, non-uniformity, and the use of non-clinically validated HPV screening methods used in different countries may contribute to a great divergence in HPV epidemiological data.

To date, HPV-targeted therapeutics are still lacking. Treatment modalities for HPV-associated cancers are either broad-spectrum antimicrobial or antiviral agents, or they are non-HPV cell-specific killing agents. Despite the high efficacy of vaccines, radiotherapy, chemotherapy, immune modulators, and antiviral agents in treating HPV-related cancers, it is unclear whether the treatment modalities can offer a complete HPV clearance, dampening viral persistency and latency. The downside of radiotherapy and chemotherapy is that these treatment regimens are non-cell type selective and may not eliminate all HPV-bearing cells. HPV DNA has been shown to be detectable in 21% of patients post-treatment with radiotherapy alone or with docetaxel, carboplatin, cisplatin and 5-fluorouracil (5-FU). This has led to recurrence, disease progression to distant metastasis, and a decreased patient survival rate [263,264,265]. In addition, vaccination and immune modulators did not seem to elicit sufficient CTL responses in HPV transgenic mice, resulting in incomplete tumour regression. These studies revealed two points: (1) vaccination and immunotherapy may not be effective in individuals predisposed to HPV infection and HPV persisted, and (2) a better animal model, e.g., humanized animal models, should be used to study treatment-related tumour regression. A recent study provided a possible explanation for HPV persistency. The presence of myeloid cells around the tumour site creates as an immunological barrier [266]. Future studies focusing on understanding the mechanism of how this pool of cells gain immune- or treatment-resistance and provide protective barriers to tumours are warranted. Furthermore, studies focusing on the oncogenic properties of under-studied HPV genotypes, like the HPV33, 39, 45, and 51 variants, of clinical importance should also be performed.

## 7. Methods

### 7.1. Literature Search Strategies

The databases used for the search of primary literature were PubMed, MEDLINE (EBSCOhost), Scopus, and Google Scholar. To ensure all relevant influential factors were included in the search strategy, the MeSH terms “human papillomavirus in Asia,” “human papillomavirus in (name of country),” “diagnosis of human papillomavirus,” “human papillomavirus screening,” “prevalence of human papillomavirus in Asia,” “HPV treatment,” “therapy for human papillomavirus cancers,” and “human papillomavirus carcinogenesis” were used in search engines.

### 7.2. Inclusion and Exclusion Criteria

We reviewed all relevant accepted publications within the recent 5 years (2015–2020). Articles that were deemed eligible and appropriate for inclusion in this review included: (1) topics covering HPV genotypes of high importance in South, Southeast, and East Asia, particularly on prevalence, HPV-associated cancers, diagnostic methods, treatment modalities, and oncogenic potential of E6- and E7-encoded by HPV; (2) articles written in English; and (3) articles with their full text accessible. There were three stages of the selection process. In the first stage, we assessed a total of 9320 articles on HPV in South, Southeast, and East Asia that were deemed appropriate for this review. Inappropriate articles (n = 8598), including articles that did not include regions and countries of interest, review articles, and meta-analysis articles, were excluded. In the second stage, we assessed the abstracts of the appropriate articles (*n* = 722). All the non-relevant and duplicate studies were rejected (*n* = 472), and the remaining 250 studies were moved to the third stage. In the third stage, we scrutinized the full content of the articles (*n* = 250), particularly the methodology and results sections. All data were arranged, grouped, and compared for their similarities and discrepancies.

## Figures and Tables

**Table 1:** Distribution of HPV genotypes in cervical cancer patients in East, Southeast, and South Asia. (**a**) A summary of HPV genotypes detected in different cervical lesions. (**b**) The prevalence of HPV genotypes in cervical patients by countries and provinces.

(a)																	
HPV Genotype			Prevalence (%)														Reference
			LSIL				HSIL				ICC			Overall			
hrHPV			48.10–91.50				81.60–99.50				64.30–100.00			53.47–100.00			[56,61,62,63,64,65,66,67,68,69,70,71,72,73,74,75,76,77,78,79,80]
16			4.70–34.30				15.00–60.10				27.30–83.78			15.56–83.78			
18			1.60–11.40				1.80–15.40				5.40–41.10			3.40–41.10			
31			1.00–8.90				0.00–10.70				0.80–5.90			1.37–8.89			
33			0.00–5.40				0.00–64.00				0.00–9.10			0.74–9.10			
35			0.00–4.30				0.00–3.00				0.10–2.60			0.50–3.20			
39			1.60–19.00				0.40–5.40				0.00–9.10			0.70–13.33			
45			0.00–2.50				0.00–3.80				0.00–9.60			0.80–9.10			
51			5.10–21.40				1.80–24.00				0.00–10.30			0.30–18.80			
52			4.50–38.00				3.50–44.60				0.00–63.60			1.08–40.74			
56			2.90–16.00				0.40–12.50				0.20–3.90			0.20–9.00			
58			5.10–21.80				1.30–27.30				0.90–24.30			1.90–15.60			
59			0.90–4.80				0.00–3.30				0.90–3.90			0.60–4.40			
68			1.40–13.90				0.20–7.10				0.00–9.10			0.40–11.11			
**(b)**																	
**Country/** **Area**	**Province/** **Region**	**Sample Size**	**HPV Genotypes (Positive Rate, %)**														**Reference**
			**hrHPV**	**HPV16**	**HPV18**	**HPV31**	**HPV33**	**HPV35**	**HPV39**	**HPV45**	**HPV51**	**HPV52**	**HPV56**	**HPV58**	**HPV59**	**HPV68**	
China	Beijing	1783	84.70	29.50	10.40	5.20	4.90	2.90	6.80	1.00	9.50	15.00	9.00	14.20	4.40	4.80	[61]
	Beijing	2817	90.10	NA	NA	NA	NA	NA	NA	NA	NA	NA	NA	NA	NA	NA	[62]
	Hunan	1336	75.70	42.50	5.10	2.50	3.90	NA	1.80	NA	NA	6.00	1.40	10.30	3.20	NA	[63]
	Guangdong	935	74.00	NA	NA	NA	NA	NA	NA	NA	NA	NA	NA	NA	NA	NA	[64]
	Yunnan	511	81.40	20.50	16.10	1.37	5.10	0.98	3.72	1.57	18.80	18.80	1.76	13.10	1.37	3.52	[65]
	Hong Kong	236	98.30	60.20	21.60	3.80	3.40	2.10	NA	3.00	0.40	11.90	0.40	9.30	NA	1.70	[66]
	NA	1337	90.10	69.50	5.60	1.90	1.30	0.50	0.70	0.80	0.30	1.40	0.20	2.20	1.20	0.40	[67]
	NA	718	74.50	47.10	41.10	1.90	1.30	NA	1.70	3.90	0.90	5.60	0.60	1.90	2.20	0.40	[68]
	NA	8343	84.37	35.48	4.78	4.29	7.90	1.31	3.28	0.87	14.77	14.77	2.62	15.13	1.57	1.97	[69]
	NA	30,636	82.80	65.60	12.60	3.50	5.50	1.20	2.20	2.10	1.40	6.50	1.30	12.60	2.60	1.30	[70]
Taiwan	NA	493	94.30	31.60	5.50	5.70	6.50	1.20	2.60	1.00	5.10	25.80	2.40	15.60	0.60	1.20	[71]
Korea	NA	1988	97.40	27.80	6.00	5.20	4.10	3.20	5.70	1.90	16.70	16.70	7.70	12.20	2.80	6.80	[72]
	NA	248	81.90	52.40	12.50	2.80	5.60	2.40	0.80	2.80	0.40	1.20	0.40	5.20	NA	0.80	[73]
Japan	NA	5045	93.50	NA	NA	NA	NA	NA	NA	NA	NA	NA	NA	NA	NA	NA	[74]
Thailand	Northern	56	94.07	15.56	5.19	8.89	0.74	0.74	13.33	1.48	8.89	40.74	5.93	8.89	2.22	11.11	[75]
	NA	11	90.90	27.30	18.20	NA	9.10	NA	9.10	9.10	NA	63.60	NA	NA	NA	9.10	[56]
Laos	Vientiane	147	53.47	23.13	3.40	2.04	1.36	1.36	NA	NA	NA	2.72	NA	10.20	NA	NA	[76]
India	Odisha	210	93.80	83.78	21.08	NA	NA	1.08	5.40	2.16	5.40	1.08	NA	2.16	NA	1.08	[77]
India	NA	128	84.38	51.56	15.63	3.13	0.78	0.78	NA	3.13	NA	3.13	NA	6.26	NA	NA	[78]
Nepal	NA	165	100.00	72.20	14.80	1.80	2.40	1.20	1.20	4.80	1.00	2.40	2.40	NA	NA	1.00	[79]
Sri Lanka	NA	106	85.85	66.04	8.49	NA	1.89	NA	NA	1.89	NA	1.89	1.89	NA	1.89	1.89	[80]

HPV: Human Papillomavirus; hrHPV: high-risk Human Papillomavirus; LSIL: low-grade squamous intraepithelial; HSIL: high-grade squamous intraepithelial; ICC: invasive cervical cancer; NA: not available.

**Table 2:** Distribution of HPV genotypes in head and neck squamous cell carcinoma (HNSCC) in East, Southeast, and South Asia. (**a**) Prevalence of different HPV genotypes detected in HNSCC. (**b**) HPV prevalence detected in different cancer stages of HNSCC. (**c**) The prevalence of HPV16 and 18 in HNSCC patients by countries.

(a)					
HPV Genotype		Overall Prevalence (%)	Reference		
16		2.60–60.70	[81,82,83,84,85,86,87,88,89,90,91,92,93,94,95,96,97,98,99,100,101,102]		
18		0.70–15.10			
31		1.60–79.20			
33		1.20			
35		0.50			
39		NA			
45		87.40			
51		NA			
52		2.00			
56		0.50–15.10			
58		NA			
59		NA			
68		49.10			
hrHPV		2.10–90.60			
**(b)**					
**Cancer Stage**		**Overall Prevalence (%)**	**Reference**		
T1		0.00–56.00	[81,82,83,84,85,86,87,88,89,90,91,92,93,94,95,96,97,98,99,100,101,102]		
T2		0.00–60.00			
T3		2.70–100.00			
T4		4.00–68.50			
N0		0.00–69.20			
N1		0.00–73.10			
N2		7.10–41.70			
N3		0.00–60.00			
**(c)**					
**Country/Area**	**Sample Size**	**HPV-Positive Rate%**	**HPV 16%**	**HPV 18%**	**Reference**
China	49	28.60	NA	NA	[83]
	303	26.40	10.60	0.70	[82]
Taiwan	100	12.00	NA	NA	[86]
Thailand	200	12.00	NA	NA	[81]
Philippines	82	2.40	NA	NA	[103]
Singapore	159	90.60	5.00	15.10	[85]
India	436	63.80	NA	NA	[87]
	88	2.60	2.60	NA	[88]
	364	13.70	9.90	1.10	[89]
	135	22.90	NA	NA	[90]
	427	2.10	1.60	NA	[84]
	43	7.00	NA	NA	[91]
	47	40.40	NA	NA	[92]
	226	29.70	14.20	NA	[93]
	31	29.00	NA	NA	[94]
	50	42.00	40.00	2.00	[95]
	250	9.20	2.80	1.60	[96]
	106	31.10	25.50	5.70	[102]
Sri Lanka	78	46.20	23.00	32.00	[97]
Pakistan	140	67.90	60.70	1.40	[98]
	100	46.00	4.00	5.00	[99]
	144	22.90	NA	NA	[100]
Bangladesh	174	21.00	19.00	NA	[101]

**Table 3:** The HPV prevalence of genotypes and different age groups of a normal population in East Asia.

Country	Area	Sample Size	HPV + %	HPV 16%	HPV 18%	HPV 31%	HPV 33%	HPV 35%	HPV 39%	HPV 45%	HPV 51%	HPV 52%	HPV 56%	HPV 58%	HPV 59%	HPV 68%	HPV 6%	HPV 11%	HPV 42%	HPV 43%	HPV 81%	0–30%	30–55%	55+ %	Reference
China	Southwest: Yunnan	28,457	12.93	1.71	0.58	0.38	0.38	0.13	0.49	0.11	0.32	2.08	0.49	1.04	0.29	0.45	0.28	0.26	0.10	NA	NA	14.96	12.07	15.04	[17]
	Southwest: Sichuan	10,682	31.50	7.40	1.89	1.15	2.56	0.59	0.28	0.32	0.67	2.28	1.79	4.46	0.87	0.73	6.58	4.73	1.05	3.77	NA	35.40	26.20	41.00	[18]
	Southwest: Guizhou	56,768	16.95	3.02	0.98	0.67	0.92	0.18	1.63	0.19	1.27	3.89	0.42	2.53	0.47	0.80	0.46	0.42	0.08	0.11	1.35	18.60	16.06	23.41	[19]
	Southwest: Chongqing	13,788	19.90	2.40	NA	NA	NA	NA	NA	NA	NA	4.80	NA	2.30	NA	NA	NA	NA	NA	NA	2.00	27.20	21.40	39.20	[20]
	South: Hainan	10,764	19.38	3.57	1.33	0.97	1.30	0.24	0.80	0.44	0.36	4.40	0.42	2.93	0.41	1.34	1.12	0.77	0.22	0.00	2.53	21.40	17.32	24.04	[21]
	South: Guangxi	9810	14.23	1.34	0.58	0.18	0.33	0.24	0.70	0.10	0.58	2.33	0.49	1.45	0.42	0.14	NA	0.26	0.34	0.60	0.75	16.10	13.50	NA	[22]
	South: Guangdong	36,871	18.34	2.95	1.07	0.37	0.75	0.36	1.18	0.25	1.00	3.33	0.88	2.09	0.70	0.37	0.69	0.46	0.45	0.82	1.06	5.81	26.54	28.91	[23]
	Northwest: Xinjiang	12,165	9.34	2.83	0.26	0.99	0.13	0.25	0.49	0.30	0.51	0.70	0.24	0.40	0.40	0.88	0.28	0.06	0.30	0.06	0.02	7.33	9.08	13.29	[24]
	North: Tianjin	2000	14.71	5.36	1.57	0.66	NA	0.05	0.56	0.25	0.05	0.66	0.40	2.22	0.35	0.40	0.40	0.51	0.10	0.15	0.20	10.81	14.86	14.50	[25]
	North: Shanxi	10,086	8.92	3.42	0.38	0.52	0.58	0.30	0.35	0.05	0.60	1.77	0.72	1.47	0.29	0.53	0.08	0.06	0.00	NA	NA	7.30	8.29	10.53	[26]
	North: Inner Mongolia	5655	14.50	5.00	1.30	1.20	0.90	0.10	1.10	0.20	1.10	1.50	0.60	2.20	0.50	0.60	0.50	0.40	0.04	0.04	0.80	13.09	14.37	15.56	[27]
	North: Hebei	26,385	33.05	8.47	2.15	2.07	2.91	0.48	2.85	0.63	2.90	5.07	1.51	5.45	0.75	1.52	2.10	1.39	2.38	0.18	0.37	34.97	30.96	37.64	[28]
	North: Beijing	21,239	21.06	4.44	1.47	1.24	0.73	0.97	2.31	0.40	2.19	4.64	2.05	4.28	1.49	1.12	NA	NA	NA	NA	NA	24.67	20.17	20.65	[29]
	East: Zhejiang	37,967	22.80	2.72	1.50	0.68	1.07	0.48	1.64	0.36	1.14	4.49	1.28	2.63	1.00	0.98	0.91	0.56	0.52	0.74	0.94	24.40	24.00	21.50	[30]
	East: Shanghai	59,541	17.92	2.85	1.01	0.97	1.28	0.29	1.46	0.29	1.45	3.58	0.57	2.64	0.40	0.99	1.07	0.91	0.23	0.11	1.94	NA	NA	NA	[31]
	East: Shandong	94,489	28.40	5.80	1.70	1.40	1.50	1.10	1.40	0.50	2.60	5.10	2.30	3.50	1.60	2.10	2.30	1.60	1.90	1.80	2.80	36.84	25.49	23.80	[32]
	East: Jiangxi	71,435	22.49	2.60	0.64	0.40	0.73	0.19	0.33	0.10	0.69	2.23	0.40	1.76	0.23	0.47	1.19	1.03	NA	0.49	NA	26.62	20.76	25.52	[33]
	East: Jiangsu	62,317	26.92	5.06	1.57	1.16	1.71	0.95	0.95	0.47	2.07	5.06	1.79	3.14	1.23	2.02	1.54	1.15	1.58	1.59	2.70	31.75	28.76	30.92	[34]
	East: Fujian	8678	38.30	8.50	2.50	2.00	2.60	0.80	2.10	0.50	1.70	7.90	1.50	6.20	1.10	2.00	2.30	2.20	0.80	0.40	3.60	38.20	38.65	51.00	[35]
	East: Anhui	19,753	16.30	7.79	0.90	0.86	1.46	0.75	0.91	0.21	1.33	4.24	1.60	3.21	0.44	1.20	0.83	0.60	NA	0.49	1.23	16.50	15.60	22.70	[36]
	Central: Hunan	12,459	20.80	2.18	0.47	0.47	0.67	0.06	0.89	0.09	0.57	3.23	0.31	2.10	0.22	0.40	0.48	0.25	0.01	0.01	0.91	25.03	19.37	18.60	[37]
	Central: Hubei	13,775	17.80	2.56	1.02	0.52	1.03	0.11	1.71	0.35	1.68	4.23	0.43	2.37	0.48	1.13	0.62	0.49	0.02	0.06	2.43	18.38	16.93	19.18	[38]
	Central: Henan	14,873	23.98	5.50	0.20	1.18	1.56	0.83	0.23	0.31	0.66	3.00	2.13	3.35	0.99	1.29	1.70	1.10	1.57	3.74	NA	28.31	23.29	26.83	[39]
	Northwest: Shaanxi	38,408	20.11	5.55	1.79	0.31	0.72	0.45	1.25	0.19	1.09	1.91	1.54	2.86	0.99	0.04	0.68	0.31	0.29	NA	NA	24.39	17.12	20.38	[40]
	Northwest: Qinghai	5892	16.36	6.31	0.78	0.83	0.61	0.03	1.32	0.04	0.46	1.36	0.17	3.45	0.25	0.56	0.34	0.39	NA	NA	0.75	1.90	15.75	26.81	[41]
	Northeast: Liaoning	6479	10.30	2.48	0.70	0.62	0.73	0.14	0.36	0.14	0.10	1.84	0.17	1.31	0.25	0.60	0.23	0.24	0.01	0.00	0.50	14.90	9.60	11.50	[42]
	Northeast: Jilin	20,648	34.40	7.80	2.40	2.20	1.92	0.76	2.81	0.73	3.31	5.80	1.93	5.00	1.45	1.75	2.34	2.03	0.53	0.35	1.91	42.80	32.20	36.60	[43]
	Northeast: Heilongjiang	24,597	32.19	5.04	1.43	1.19	1.43	1.14	1.10	0.59	2.31	4.34	2.20	2.99	1.91	1.75	1.91	1.28	2.04	1.98	2.76	34.96	13.11	31.88	[44]
	Hong Kong SAR	108	41.70	6.50	1.90	2.80	NA	NA	1.90	NA	NA	4.60	4.60	2.80	3.70	NA	0.90	0.90	NA	2.80	1.90	44.00	37.87	52.90	[45]
Korea	South Korea	968	40.60	4.50	1.80	0.52	0.83	0.83	0.94	0.11	3.50	6.10	2.50	4.80	NA	4.20	1.00	0.30	3.60	2.80	NA	NA	NA	NA	[46]
	South Korea	18,170	16.71	1.39	0.69	0.47	0.31	0.64	1.33	0.39	0.98	1.85	0.96	1.87	0.35	1.17	0.56	0.21	0.90	0.82	NA	NA	NA	NA	[47]
	South Korea	18,815	27.80	2.00	1.00	1.10	0.90	1.20	1.30	0.40	1.80	3.20	1.90	2.70	0.80	3.00	0.80	0.10	2.00	1.40	NA	36.10	26.92	29.57	[48]
Mongolia	Ulaanbaatar	100	47.00	21.00	2.00	5.00	6.00	2.00	3.00	4.00	1.00	14.00	2.00	5.00	4.00	1.00	NA	NA	1.00	NA	3.00	63.00	46.40	36.00	[50]
Japan	NA	770	16.20	3.10	0.90	1.20	NA	0.10	0.20	0.10	1.70	6.40	3.00	2.60	1.00	1.20	NA	NA	NA	NA	NA	NA	NA	NA	[51]
	NA	3047	25.00	3.40	1.00	1.50	0.30	0.40	1.90	0.30	2.10	5.30	2.10	2.60	0.70	1.20	0.90	0.20	1.50	NA	NA	NA	NA	NA	[49]

**Table 4:** The positive detection rate of HPV prevalence in cervical lesions by countries and age groups in Southeast Asia.

Country	Area	Sample Size	HPV Genotypes (Positive Rate, %)																			Age Group (Positive Rate, %)			Reference
			All HPV	HPV16	HPV18	HPV31	HPV33	HPV35	HPV39	HPV45	HPV51	HPV52	HPV56	HPV58	HPV59	HPV68	HPV6	HPV11	HPV42	HPV43	HPV81	0–30	30–55	55+	
Indonesia	NA	78	17.90	1.30	15.40	NA	NA	NA	NA	NA	NA	NA	NA	NA	NA	NA	NA	NA	NA	NA	NA	14.10	64.20	21.80	[52]
Malaysia	Sabah	240	9.60	0.80	NA	NA	0.40	NA	NA	NA	NA	NA	1.70	0.80	0.40	NA	NA	NA	NA	NA	0.40	14.30	8.80	14.30	[53]
	NA	394	39.10	14.00	10.20	0.80	1.80	0.30	NA	0.80	NA	0.50	NA	3.60	NA	1.00	NA	NA	NA	NA	1.00	NA	NA	NA	[54]
	NA	1190	7.20	1.30	0.60	0.60	NA	NA	NA	0.70	NA	1.00	NA	0.90	NA	NA	NA	NA	NA	NA	NA	7.60	7.00	7.30	[55]
Thailand	NA	1250	24.60	2.10	0.70	0.60	0.60	0.20	1.60	0.20	1.80	3.60	0.50	0.90	0.90	1.70	0.10	0.10	0.50	NA	0.70	49.10	48.30	NA	[56]
	NA	5906	15.10	1.40	0.60	0.30	0.20	0.10	0.60	0.10	0.90	1.60	0.30	0.80	0.30	0.60	0.20	0.10	0.50	NA	0.40	24.80	31.30	22.00	[57]

**Table 5:** The positive detection rate of HPV prevalence in cervical lesions by countries and age groups in South Asia.

Country	Area	Sample Size	HPV Genotypes (Positive Rate, %)																			Age Group (%)			Reference
			All HPV	HPV16	HPV18	HPV31	HPV33	HPV35	HPV39	HPV45	HPV51	HPV52	HPV56	HPV58	HPV59	HPV68	HPV6	HPV11	HPV42	HPV43	HPV81	0–30	30–55	>55	
Nepal	NA	1289	14.40	0.80	2.30	0.90	0.60	0.00	0.20	0.40	1.20	0.10	0.20	0.70	1.10	0.20	0.60	0.20	2.20	0.60	NA	14.30	15.00	12.90	[58]
	NA	998	19.70	6.70	1.20	0.60	2.60	0.30	4.80	0.10	1.40	0.40	0.80	2.80	0.00	0.30	0.60	0.30	0.00	0.00	0.40	14.40	9.20	30.00	[59]
Sri Lanka	NA	483	44.40	5.00	NA	NA	NA	NA	NA	NA	NA	NA	NA	NA	NA	NA	9.40	NA	NA	NA	NA	NA	NA	NA	[60]

**Table 6:** The commercial HPV nucleic acid detection systems used in East, Southeast, and South Asia.

Region	Country/Area	Detection Method/Company	HPV Type Detection	Technology	References
East, South, and Southeast Asia	China, Thailand, Vietnam, Malaysia, and Nepal	HPV GenoArray Diagnostic Kit (Hybribio, Chaozhou, China)	21 HPV genotypes:	PCR-flow through hybridization fluorescence and gene chip system	[59,115,151,152,153,154,155,156]
			13 hrHPV: HPV16, 18, 31, 33, 35, 39, 45, 51, 52, 56, 58, 59, and 68);		
			2 intermediate-risk HPV: HPV53 and 66;		
			6 lrHPV: HPV6, 11, 42, 43, 44, and 81		
	South Korea, Malaysia, and Nepal	Anyplex™ II HPV 28 (Seegene, Seoul, Korea)	28 HPV genotypes: 13 hrHPV: HPV16, 18, 31, 33, 35, 39, 45, 51, 52, 56, 58, 59, and 68; 5 intermediate-risk HPV: HPV26, 53, 66, 70, and 82; 8 lrHPV: HPV6, 11, 40, 42, 43, 44, 54, 61, 69, and 73	Quantitative RT-PCR	[47,105,157]
East Asia	China	HPV Geno-Array test kit (Genetel Pharmaceuticals Co, Ltd., Shenzhen, China)	26 HPV genotypes:	PCR-flow through hybridization and gene chip system	[119,158]
			13 hrHPV: HPV16, 18, 31, 33, 35, 39, 45, 51, 52, 56, 58, 59, and 68;		
			3 intermediate-risk HPV: HPV53, 66, and 67;		
			10 lrHPV: HPV6, 11, 40, 42, 43, 44, 54, 55, 67, and 73		
		HPV Genotyping Panel kit (TELLGEN Life Science Co. Ltd., Shanghai, China)	27 HPV genotypes:	PCR-flow through hybridization fluorescence and gene chip system	[159]
			13 hrHPV: HPV16, 18, 31, 33, 35, 39, 45, 51, 52, 56, 58, 59, and 68;		
			4 intermediate-risk HPV: HPV26, 53, 66, and 82;		
			10 lrHPV: HPV6, 11, 40, 42, 43, 44, 55, 61, 81, and 83		
		Human Papillomavirus Genotyping Detection Kit (Microarray) (Crystal Core^®^, CapitalBio Corporation, Beijing, China)	22 HPV genotypes:	PCR-flow through hybridization fluorescence and gene chip system	[59,160]
			13 hrHPV: HPV16, 18, 31, 33, 35, 39, 45, 51, 52, 56, 58, 59,and 68;		
			4 intermediate-risk HPV: HPV26, 53, 66, and 82;		
			4 lrHPV: HPV6, 11, 70, and 81		
		HPV nucleic acid amplification typing test kit (Kaipu Biochemical Company, Chaozhou, Guangdong, Korea)	21 HPV genotypes	PCR-flow through hybridization fluorescence and gene chip system	[121]
			13 hrHPV: HPV 16, 18, 31, 33, 35, 39, 45, 51, 52, 56, 58, 59, and 68;		
			2 intermediate risk HPV: HPV53 and 66;		
			6 lrHPV: HPV 6, 11, 42, 43, and 44, as well as CP8304		
		HPV genotyping kit (bioPerfectus Technologies, Jiangsu, China)	21 HPV genotypes:	Fluorescence-based multiplex PCR	[148]
			13 hrHPV: HPV 16, 18, 31, 33, 35, 39, 45, 51, 52, 56, 58, 59,and 68;		
			2 intermediate-risk HPV: HPV53 and 66;		
			3 lrHPV: HPV6, 11, and 81		
		PCR-RDB assay (Yaneng Bioscience Co. Ltd., Shenzhen, China)	23 HPV genotypes:	PCR and reverse dot blot assay	[33,83,127,155,161,162]
			13 hrHPV: HPV 16, 18, 31, 33, 35, 39, 45, 51, 52, 56, 58, 59, and 68;		
			3 intermediate-risk HPV: HPV53, 66, and 82;		
			7 lrHPV: HPV6, 11, 42, 43, 73, and 81		
		HPV Genotyping real-time PCR kit (Zhejiang Bio-Tech Co. Ltd., Shanghai, China)	15 HPV genotypes: 13 hrHPV: HPV16, 18, 31, 33, 35, 39, 45, 51, 52, 56, 58, 59, and 68; 2 intermediate-risk HPV: HPV66 and 82	Real-time PCR	[62,163,164]
		SPF10 PCR-DEIA-LiPA25 (Labo Biomedical Products, Rijswijk, The Netherlands)	25 HPV genotypes:	DNA enzyme immunoassay (DEIA), reverse hybridization and multiplex PCR	[165]
			13 hrHPV: HPV16, 18, 31, 33, 35, 39, 45, 51, 52, 56, 58, 59, and 68;		
			3 intermediate-risk HPV: HPV53, 66 and 70;		
			9 lrHPV: HPV6, 11, 34, 40, 42, 43, 44, 54, and 74		
	Hong Kong SAR	GP5+/GP6+_52HK (modified GP5+/6+ L1); HPV16 and 18 E6*I primer probes; primers targeting E7 and L1	2 hrHPV: HPV16 and 18	Immunostaining; PCR, qRT-PCR and ddPCR	[120]
		L1 ORF	Full spectrum of HPV genotypes (*Alpha*-, *Beta*-, and *Gamma*-HPV)	PCR-based next generation sequencing (NGS) assay	[166]
	Hong Kong SAR and Macao SAR	PGMY09/11 primers and SNIPER^®^ HPV Genotyping Diagnosis Kit (Genetel, Shenzhen, China)	29 HPV genotypes:	PCR and fluorescence probes	[131]
			13 hrHPV: HPV 16, 18, 31, 33, 35, 39, 45, 51, 52, 56, 58, 59, and 68;		
			6 intermediate-risk HPV: HPV26, 53, 66, 69, 82, and 67;		
			10 lrHPV: HPV6, 11, 40, 42, 43, 44, 54, 55, 57, and 73		
	Taiwan	EasyChip HPV Blot genotyping array (King Car Biotechnology Co., Ltd., Yilan County, Taiwan)	38 HPV genotypes:	PCR-RDB	[167,168]
			13 hrHPV: HPV 16, 18, 31, 33, 35, 39, 45, 51, 52, 56, 58, 59, and 68;		
			8 intermediate-risk HPV: HPV26, 53, 66, 67, 69, 70, 82, and 85;		
			17 lrHPV: HPV6, 11, 32, 37, 42, 43, 44, 54, 55, 61, 62, 71, 72, 74, 81, 83, and 84		
	South Korea	MyHPV Chip Kit^®^, BioMedLab, Seoul, Korea	24 HPV genotypes: 13hrHPV: HPV 16, 18, 31, 33, 35, 39, 45, 51, 52, 56, 58, 59, and 68; 3 intermediate-risk HPV: HPV53, 66, and 70; 8 lrHPV: HPV6, 11, 34, 40, 42, 43, 44, 54, and 70	PCR chip microarray	[169]
		HPV Genotyping Chip^™^ Kit (AGBIO Diagnostics, Seoul, Korea)	32 HPV genotypes: 13hrHPV: HPV 16, 18, 31, 33, 35, 39, 45, 51, 52, 56, 58, 59, and 68; 5 intermediate-risk HPV: HPV26, 53, 66, 69, and 70; 14 lrHPV: HPV6, 11, 32, 34, 40, 42, 43, 44, 54, 55, 57, 61, 62, and 73	PCR chip microarray	[148]
		Cheil HPV DNA chip kit (Cheil General Hospital, Seoul, Korea)	35 HPV genotypes: 13 hrHPV: HPV 16, 18, 31, 33, 35, 39, 45, 51, 52, 56, 58, 59, and 68; 6 intermediate-risk HPV: 30, 53, 66, 67, 69, and 70; 16 lrHPV: HPV6, 11, 32, 40, 42, 43, 44, 54, 55, 62, 72, 81, 82, 84, 90, and 91	SYBR Green qRT-PCR and microarray	[72]
		HPV Liquid Bead Microarray (Osang Healthcare, Anyang, China)	32 HPV genotypes: 13 hrHPV: HPV 16, 18, 31, 33, 35, 39, 45, 51, 52, 56, 58, 59, and 68; 5 intermediate-risk HPV: HPV26, 53, 66, 69, and 70; 14 lrHPV: HPV6, 11, 32, 34, 40, 42, 43, 44, 54, 55, 62, 73, 81, and 83	PCR chip microarray	[47]
		HPV 9G DNA chip (Biometrix Technology Inc., Chuncheon, Korea)	38 HPV genotypes: 13 hrHPV: HPV16, 18, 31, 33, 35, 39, 45, 51, 52, 56, 58, 59, and 68; 6 intermediate-risk HPV: HPV26, 53, 66, 67, 69, and 70; 19 lrHPV: HPV3, 6, 10, 11, 27, 32, 34, 40, 42, 43, 44, 54, 55, 57, 61, 62, 71, 73, and 74	PCR chip microarray	[170]
		GeneFinder HPV Liquid Bead MicroArray Genotype kit (GeneFinder; Infopia Inc., Anyang, China)	32 HPV genotypes: 13 hrHPV: HPV16, 18, 31, 33, 35, 39, 45, 51, 52, 56, 58, 59, and 68; 5 intermediate-risk HPV: HPV26, 53, 66, 69, and 70; 14 lrHPV: HPV6, 11, 32, 34, 40, 42, 43, 44, 54, 55, 62, 73, 81, and 83	Microsphere bead–based PCR and hybridisation	[170]
	Japan	Genosearch-31 (GS-31) ((Medical & Biological Laboratories, Co., Ltd. (MBL), Nagoya, Japan)	31 HPV genotypes: 13 hrHPV: HPV16, 18, 31, 33, 35, 39, 45, 51, 52, 56, 58, 59, and 68; 5 intermediate-risk HPV: HPV26, 53, 66, 70, and 82; 13 lrHPV: HPV6, 11, 42, 44, 54, 55, 61, 62, 71, 73, 84, 89, and 90	PCR-SSOP-Luminex system	[51,171,172]
		HPV Thirteen (Nihon Gene Research Laboratories Inc., Sendai, Japan)		PCR	[130]
East and Southeast Asia	Hong Kong SAR and Malaysia	GeneFlow HPV array test (DiagCor Bioscience Inc., Hong Kong SAR, China)	33 HPV genotypes: 13 hrHPV: HPV16, 18, 31, 33, 35, 39, 45, 51, 52, 56, 58, 59, and 68; 5 intermediate-risk HPV: HPV26, 53, 66, 70, and 82; 15 lrHPV: HPV6, 11, 40, 42, 43, 44, 54, 55, 57, 61, 71, 72, 73, 81, and 84	PCR-RDB assay and rapid flow through hybridization assay	[54,173]
South East Asia	Thailand	AmoyDx^®^ Human Papillomavirus Genotyping Detection Kit from Amoy Diagnostics, Xiamen, China)	21 HPV genotypes: 13 hrHPV: HPV16, 18, 31, 33, 35, 39, 45, 51, 52, 56, 58, 59, and 68; 5 intermediate-risk HPV: HPV26, 53, 66, 79, and 82; 3 lrHPV: HPV6, 11, and 73	Real-time PCR	[174]
		DNA ISH HPV III Family Probe (Ventana Medical Systems, Tucson, Arizona, USA)	12 HPV genotypes:11 hrHPV: HPV16, 18, 31, 33, 35, 39, 45, 51, 52, 56, and 58;1 intermediate-risk HPV: HPV66	Immunohistochemistry and in situ hybridization	[174]
		Reverse line blot hybridization (RLBH) developed by van de Brule and co-workers	35 HPV genotypes: 13 hrHPV: HPV16, 18, 31, 33, 35, 39, 45, 51, 52, 56, 58, 59, and 68; 5 intermediate-risk HPV: HPV26, 53, 66, 70, and 82; 17 lrHPV: HPV6, 11, 34, 40, 42, 43, 44, 54, 55, 57, 61, 71, 72, 73, 81, 83, and 84	PCR and reverse line blot hybridization	[153,175]
		HPV OncoTect^®^ E6, E7 mRNA Kit(IncellDx, Menlo Park, CA, USA)	13 hrHPV: HPV16, 18, 31, 33, 35, 39, 45, 51, 52, 56, 58, 59, and 68	Reverse transcription and flow cytometry	[176]
	Singapore	Multiplex luminescence ELISA	8 hrHPV: HPV16, 18, 31, 33, 35, 45, 52, and 58	Fluorescence-labelled GST-E6/E7/L1 fusion proteins and ELISA	[177]
South Asia	India	TS-E7-MPG (developed by Schmitt and colleagues, in IARC, Lyon, France)	22 HPV genotypes: 13 hrHPV: HPV16, 18, 31, 33, 35, 39, 45, 51, 52, 56, 58, 59, and 68; 6 intermediate-risk HPV: HPV26, 53, 66, 67, 70, and 82; 3 lrHPV: HPV6, 11, and 73	Luminex-based multiplex-type-specific E7 PCR	[178]
	Sri Lanka	PapType hrHPV detection and genotyping kit (Genera Biosystems Limited, Australia)	16 HPV genotypes: 13 hrHPV: HPV16, 18, 31, 33, 35, 39, 45, 51, 52, 56, 58, 59, and 68; 1 intermediate-risk HPV: HPV66; 2 lrHPV: HPV6 and 11	Flow cytometry	[60]

## Data Availability

Not applicable.
